# Seroepidemiological surveillance of *Burkholderia pseudomallei* in Bangladesh

**DOI:** 10.1016/j.trstmh.2012.06.003

**Published:** 2012-09

**Authors:** Rapeephan R. Maude, Richard J. Maude, Aniruddha Ghose, Md Robed Amin, Md Belalul Islam, Mohammad Ali, Md Shafiqul Bari, Md Ishaque Majumder, Vanaporn Wuthiekanan, Arjen M. Dondorp, Robin L. Bailey, Nicholas P.J. Day, M. Abul Faiz

**Affiliations:** a Mahidol–Oxford Tropical Medicine Research Unit (MORU), Faculty of Tropical Medicine, Mahidol University, 3/F, 60th Anniversary Chalermprakiat Bldg, 420/6 Rajvithi Road, Rajathevi, Bangkok 10400, Thailand; b Centre for Tropical Medicine, CCVTM, Nuffield Department of Clinical Medicine, Churchill Hospital, Oxford, UK; c Chittagong Medical College, Chittagong, Bangladesh; d Dhaka Medical College, Dhaka, Bangladesh; e Comilla Medical College, Comilla, Bangladesh; f Shaheed Ziaur Rahman Medical College, Bogra, Bangladesh; g MAG Osmani Medical College, Sylhet, Bangladesh; h Sir Salimullah Medical College, Dhaka, Bangladesh; i London School of Hygiene & Tropical Medicine, Keppel Street, London, UK

**Keywords:** *Burkholderia pseudomallei*, Melioidosis, Indirect haemagglutination assay, Seroprevalence, Epidemiological surveillance, Bangladesh

## Abstract

Melioidosis (*Burkholderia pseudomallei* infection) has yet to be demonstrated systematically in Bangladesh. A prospective, cross-sectional serological survey was conducted in 2010 at six Bangladeshi hospitals. Age, gender, occupation and residential address were recorded. Of 1244 patients, 359 (28.9%) were positive for *B. pseudomallei* by indirect haemagglutination assay. Farmers had an increased risk of seropositivity (risk ratio = 1.4, 95% CI 1.0–1.8; p = 0.03). There was no clear geographic clustering of seropositives. Melioidosis should be considered as a possible cause of febrile illness in Bangladesh. Further studies are needed to establish the incidence of clinical disease and distribution of environmental risk.

## 1 Introduction

Melioidosis is caused by *Burkholderia pseudomallei*, an intracellular, Gram-negative bacterium present in wet soil and rice paddies throughout the tropics, particularly in the rainy season, and is highly endemic in Southeast Asia and northern Australia.^1^ The incidence of clinical melioidosis is strongly associated with the degree of exposure to the organism.^1^ Bangladesh has large areas of rice paddy fields, a tropical climate and heavy monsoon rains for around 6 months each year with frequent and severe flooding. There have been a few case reports of melioidosis in patients from Bangladesh visiting or staying in other countries.^2^, ^3^ The extent of exposure to *B. pseudomallei* and the incidence of clinical melioidosis in Bangladesh are unknown. There is a lack of confirmatory diagnostic facilities and a low index of suspicion among clinicians. Therefore, a hospital-based seroprevalence study was conducted to quantify exposure to *B. pseudomallei* in unselected patients from across Bangladesh.

## 2 Materials and methods

### 2.1 Patient recruitment

Patients were recruited between June and August 2010 at Chittagong Medical College, Dhaka Medical College, Sir Salimullah Medical College (Dhaka), Comilla Medical College, Bogra Medical College and Sylhet Medical College hospitals in Bangladesh. These are government tertiary-care hospitals with very large catchment areas covering five of the seven Divisions of Bangladesh. Entry criteria were patients of all ages and both genders presenting to hospital, providing written informed consent and having a blood test for another purpose from which remaining serum or plasma would be available for the study. Age, gender, area of residence and occupation were recorded.

### 2.2 Laboratory techniques

Antibody levels to *B. pseudomallei* were quantified using the indirect haemagglutination assay (IHA). The methodology for this has been described in detail elsewhere.^4^ This study used standard pooled antigens that were separately prepared from two *B. pseudomallei* isolates from Thai melioidosis patients (strains 199a and 207a). The cut-off for low seropositivity was an antibody titre of ≥1:10 and for high seropositivity was ≥1:160.^5^

### 2.3 Statistical analysis

Statistical analysis was done using STATA 11/SE (StataCorp LP, College Station, TX, USA). Univariate group comparisons were performed using χ^2^ and Fisher's exact tests. Associations of antibody titre with age were determined using linear regression by the least squares method. Statistical significance was set at the 5% level.

## 3 Results

Of 1250 patients enrolled in the study, 6 patients were excluded due to inadequate specimens for analysis. The median age of patients was 40 years (range 1–104 years), of which 64 (5.1%) were <16 years old and 7 (0.6%) were <5 years old. Moreover, 682 (54.8%) of the 1244 patients were male. The commonest occupations were housewife (37.5%), farmer (15.4%) and service industry worker (15.2%); 56% were from rural areas.

Of 1244 patients, 359 (28.9%) were seropositive for *B. pseudomallei* (titre ≥1:10) and 43 (3.5%) had high-titre seropositivity (≥1:160). Farmers were more likely to be seropositive with the low cut-off (≥1:10) (risk ratio = 1.4, 95% CI 1.0–1.8; p = 0.03), whilst males had a higher risk of seropositivity with the high cut-off (≥1:160) (risk ratio = 1.3, 95% CI 1.1–1.6; p = 0.05). There was no correlation between the proportion seropositive and age (p = 0.60). There was no significant difference in seropositivity between people from urban and rural areas. Regarding area of residence, 45% of patients from Chittagong, 33% from Bogra, 26% from Sylhet, 24% from Dhaka and 18% from Comilla Division were seropositive; 5% of patients from Chittagong, 2% each from Sylhet and Comilla, and 1% each from Bogra and Dhaka had a high antibody titre (≥1:160).

## 4 Discussion

Approximately one-third of patients in this study had evidence of exposure to *B. pseudomallei*. This is much higher than expected from the low reported incidence of clinical cases and low seropositivity rates elsewhere in the region.^1^ The clinical presentation of melioidosis is non-specific. Unless it is specifically sought by clinicians it can be easily overlooked. In Thailand, an antibody titre of ≥1:160 is commonly used to support a diagnosis in those with clinical features,^5^ although serological testing per se has low specificity in highly endemic areas. The highest seropositivity rate in this study was in Chittagong Division where almost one-half of the participants were seropositive and 5% had high antibody titres. This is comparable with high antibody titres in low-endemic parts of Thailand (7–10%) and Myanmar (7%).^5^ In contrast, highly endemic areas in Thailand where melioidosis is the leading cause of sepsis have seropositivity rates of approximately 60–80% with high antibody titres in around one-third.^5^

The limitations of this study were that it was not done in a healthy population and that children (<16 years) were under-represented, which might cause an overestimate of the overall seropositivity rate. The IHA test used can also be positive due to *B. thailandensis*, a non-pathogenic organism commonly found in Thailand.^1^ Thai isolates were used for the IHA test^5^ as there are no such isolates from Bangladesh. The study did not collect information on clinical disease or risk factors for melioidosis in the study group.

This study has newly identified serological evidence of exposure to *B. pseudomallei* as being relatively common in Bangladesh. It is not known how this relates to the possible burden of clinical disease. If the incidence of clinical disease is as high as might be predicted from this study, this has important implications for local empirical treatment guidelines. Further studies are required to investigate the presence of the organism in soil and to determine the epidemiology, incidence and spectrum of clinical disease in Bangladesh.

## Authors’ contributions

RRM, RJM, VW, AG, MRA, MBI, MA, MSB, MIM and MAF conceived the study; RRM, RJM, VW, AG and MAF designed the study; RRM, RJM and VW analysed and interpreted the data; AMD, RLB and NPJD contributed to interpretation of the data; RRM, RJM and NPJD drafted the manuscript. All authors revised the manuscript critically for intellectual content, and read and approved the final version. NPJD is guarantor of the paper.

## Funding

The study was funded by the Wellcome Trust of Great Britain (London, UK) (grant no. B9RPYY0) and the London School of Hygiene & Tropical Medicine (London, UK) (MSc summer projects funding no. 491863).

## Competing interests

None declared.

## Ethical approval

Ethical approval for this study was obtained from the Bangladesh Medical Research Council Ethics Committee, the London School of Hygiene & Tropical Medicine Ethics Committee (UK) and the Oxford Tropical Research Ethics Committee (OXTREC).
